# Independent Adipogenic and Contractile Properties of Fibroblasts in Graves’ Orbitopathy: An *In Vitro* Model for the Evaluation of Treatments

**DOI:** 10.1371/journal.pone.0095586

**Published:** 2014-04-21

**Authors:** He Li, Caroline Fitchett, Katarzyna Kozdon, Hari Jayaram, Geoffrey E. Rose, Maryse Bailly, Daniel G. Ezra

**Affiliations:** 1 Department of Cell Biology, UCL Institute of Ophthalmology, London, United Kingdom; 2 Department of Ocular Biology and Therapeutics, UCL Institute of Ophthalmology, London, United Kingdom; 3 Moorfields Eye Hospital, and the National Institute for Health Research (NIHR) Biomedical Research Centre at Moorfields Eye Hospital NHS Foundation Trust and UCL Institute of Ophthalmology, UCL Partners AHSC, London, United Kingdom; University of Bergen, Norway

## Abstract

Graves’ orbitopathy (GO) is a disfiguring and sometimes blinding disease, characterised by inflammation and swelling of orbital tissues, with fibrosis and adipogenesis being predominant features. Little is known about the disease aetiology and the molecular mechanisms driving the phenotypic changes in orbital fibroblasts are unknown. Using fibroblasts isolated from the orbital fat of undiseased individuals or GO patients, we have established a novel *in vitro* model to evaluate the dual profile of GO cells in a three-dimensional collagen matrix; this pseudo-physiological 3D environment allows measurement of their contractile and adipogenic properties. GO cells contracted collagen matrices more efficiently than control cells following serum or TGFβ1 stimulation, and showed a slightly increased ability to proliferate in the 3D matrix, in accordance with a fibro-proliferative phenotype. GO cells, unlike controls, also spontaneously differentiated into adipocytes in 3D cultures - confirming an intrinsic adipogenic profile. However, both control and GO cells underwent adipogenesis when cultured under pathological pressure levels. We further demonstrate that a Thy-1-low population of GO cells underlies the adipogenic - but not the contractile - phenotype and, using inhibitors, confirm that the contractile and adipogenic phenotypes are regulated by separate pathways. In view of the current lack of suitable treatment for GO, we propose that this new model testing the duality of the GO phenotype could be useful as a preclinical evaluation for the efficacy of potential treatments.

## Introduction

Graves’ Orbitopathy (GO) is a common manifestation that affects up to 50% of patients with autoimmune thyroid disease [1]. The morbidity of GO is largely related to orbital fat expansion, this resulting from several pathological processes including adipogenesis, hyaluronan secretion and fibrosis [2]–[4]. Whilst the specificity of these changes to orbital tissues remains poorly understood, GO orbital fibroblasts have been shown to exhibit distinctive Thy-1 [5], [6], CD34 [7] and IGF-1 receptor (IGF-1R) [8] profiles, as well as unique responses to epigenetic factors such as enhanced chemokine production, adipogenesis and hyaluronan secretion [3], [9]. Thy-1 expression is of particular interest, as it was shown that segregation of fibroblasts on the basis of Thy-1 expression reflects differences in cell fate - with only Thy-1 negative cells being able to undergo adipogenesis, a key pathological feature of GO [10]. Thy-1 expression has been shown to be attenuated in GO fibroblasts, possibly underlying a pro-adipogenic phenotype [5], [6], [10].

In addition to the distinctive cell types, there are also unique anatomical considerations in the orbit that might mediate site-specific influences. The orbit is a conical compartment, enclosed by bony wall and a tough anterior orbital septum [11], and any increase in tissue volumes resulting from inflammatory oedema or venous congestion can lead to a marked rise in intraorbital pressure. Direct manometry has shown intraorbital pressure to rise from 4 mmHg in normal orbits [12] to 27 mmHg in severe GO [13]. Tissue mechanics is a fundamental process governing cell proliferation, migration and differentiation [14], [15]. Although tissue tension is known to modulate stem cell differentiation, and particularly adipogenesis [16], nothing is known about the mechanobiology of GO, despite marked changes in the mechanical environment of GO fibroblasts during the course of the disease.

We hypothesised that the disordered mechanical environment in active GO might underlie some aspects of the pathogenesis of this condition. We here demonstrate, using a novel *in vitro* 3D culture model, that reproducing a physiological environment induces a spontaneous Thy-1-dependent adipogenesis in GO fibroblasts. We also show that GO fibroblasts, as compared to those from undiseased orbits, are more contractile in a 3D functional model of fibrosis, and that this difference is not linked to Thy-1 expression. Finally, we describe how our 3D model can be used to interrogate potential pathways mediating adipogenesis and fibrosis and putatively evaluate new treatments for GO.

## Materials and Methods

### Ethics Statement

This study adhered to the tenets of the Declaration of Helsinki and was approved by the National Research Ethics Service Committee London- Bentham (REC reference 11/LO/1170). The study was explained to potential study participants and written informed consent was obtained before enrolment.

### Clinical Samples

Orbital fat was harvested from 3 patients with active GO undergoing orbital decompression and from 3 control patients undergoing removal of subconjunctival fat herniation. The clinical features of these patient groups are presented in Table 1. The biopsies were mechanically dispersed and the tissue fragments placed in tissue culture dishes in Dulbecco’s modified Eagle’s medium (DMEM) with 4.5g/L l-Glutamine (PAA), supplemented with 10% foetal bovine serum (FBS, Sigma), 100 IU/ml penicillin, 100 µg/ml streptomycin (Invitrogen) at 37°C with 5% carbon dioxide. Following growth from the explant, the fibroblast populations (controls: CO2, CO3, CO4; GO populations: HO1, HO2, HO3) were trypsinized and maintained routinely in the above medium. The fibroblast populations were found to be positive for the mesenchymal marker vimentin, and negative for cytokeratin and Factor VIII (Methods S1 and Figure S1), as well a negative for the fibrocyte marker CD45 (Figure S2), confirming their fibroblast nature [6] [10]. The cells were used between passage 4 and 9 for all experiments.

**Table 1:** Origin of the samples of orbital fibroblasts.

Cell line	Age	Gender	Condition	Duration of GO/months	CAS Score
CO2	65	M	Orbital fat Prolapse	n/a	n/a
CO3	68	F	Orbital fat Prolapse	n/a	n/a
CO4	49	M	Evisceration	n/a	n/a
HO1	71	F	GO	15	6
HO2	60	M	GO	8	5
HO3	57	M	GO	6	6

### Collagen Contraction Assay

The collagen contraction assays were performed as previously described [17]. GO or control cells were seeded in a 1.5 mg/ml collagen type-I matrix (First Link (UK), Ltd) at a concentration of 7×10^4^ cells/ml, within the coverslip area of a 35 mm diameter MatTek dish (MatTek Corporation, Ashland). Following polymerisation, the gels were detached from the edge of the well, 2 ml of culture medium were added, and gel contraction was monitored daily for 7 days by digital photography. Gel surface area was measured using ImageJ software (http://rsb.info.nih.gov/ij/), and the contraction expressed as the percentage decrease in gel area with respect to the original area (day 0). For cytokine stimulation, the gels were made with 14×10^4^ cells/ml in serum free medium with/without 5 ng/ml recombinant human TGFβ1 or 10 ng/ml IL1β (R&D systems). For the testing of inhibitors, 10 µM Imatinib (Cayman Chemicals), 10 µM PP2 (Tocris Bioscience) or 5 µg/ml 1H7 (anti-IGF1R antibody, BioLegend) were added to the medium at day 0 and maintained throughout the assay. For 1H7, the antibody was also incorporated into the gel mix before polymerization, at a final concentration of 10 µg/ml.

AlamarBlue (Invitrogen, Carlsbad, California) was used to measure the relative proliferation of the fibroblasts in the collagen matrices. A 10% v/v Alamar Blue solution was mixed with 2 ml of the gel contraction assay culture medium at day 0 (1 our after gel setting), day3 and day 7 and incubated for 24 h. The fluorescence signal of sample medium from each well was read using peak excitation and emission wavelengths of 544 nm and 590 nm respectively (Fluostar Optima, BMG Biotech). Each experiment was carried out in triplicate and repeated on at least 3 separate occasions.

### Adipogenic Differentiation in Monolayers (2D)

After plating 1×10^5^ cells into 35 mm dishes with growth medium, the cells were allowed to become confluent and maintained at 100% confluency for 2 days. The medium was then replaced with Adipocyte Differentiation Medium (ADM, Zenbio) for a further 3 days, after which the medium was changed to Maintenance medium and the cultures maintained for a further 2 weeks. Adipogenic differentiation was assessed by Oil-Red-O staining. Briefly, the cell monolayers were washed in PBS and fixed for 20 minutes with 10% formalin (Sigma). The monolayers were washed twice in PBS and once in distilled water, and incubated with 60% isopropanol for a further 5 minutes. An Oil-Red-O staining solution was prepared as a 3 mg/ml stock in 99% isopropanol, diluted 3∶2 v/v in distilled water, and filtered immediately prior to use. After removal of the isopropanol, the cells were stained with the diluted Oil-Red-O solution for 2 minutes and washed in distilled water until the water ran clear. Haematoxylin was added for 1 minute, and the cells washed again in water until it ran clear. Evidence of adipocyte differentiation (red lipid droplets) was observed with a 10x oil-objective on a Zeiss Axioplan 200 microscope, the images acquired using the attached Axioplan MRC camera, and processed in with Adobe Photoshop software for matching brightness and contrast.

### Adipogenic Differentiation in Gels (3D) and Application of External Pressure

**Figure 1:**
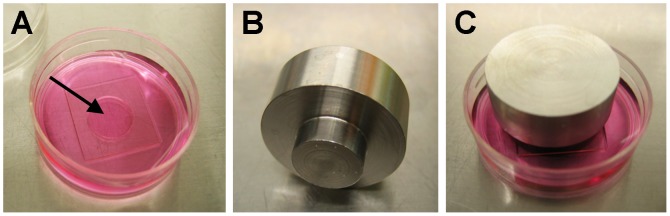
3D pressure set up. Fibroblasts were embedded into a collagen gel within the well of a Mattek dish (A) and allowed to establish in the gel for 5 days. Pressure equivalent to pathological levels in GO (28 mmHg) was then applied to the gel by laying a coverslip on top and placing a weight (B) on it. The pressure set up (C) was maintained for 48 hours and the gels were fixed and processed for staining or RNA extraction.

MatTek dishes were washed for 2 minutes with 1 M HCl, followed by a rinse in 70% ethanol, and a rinse in PBS. The coverslip area was then coated with 400 µg/ml collagen for 15 minutes, the excess collagen aspirated, and the area allowed to dry. GO or control cells were seeded into collagen gels within the well area as for the contraction assay, but the gels were not detached after polymerization, and 2 ml of medium was carefully added to the dish so as to not disturb the gel area. The attached gels were cultured for 5 days, and further left untreated or subjected to pressure for another 48 hours. An equivalent of 28 mmHg pressure was generated by layering the gels with a fine sterile fabric mesh, a 13 mm diameter coverslip, and a custom machined stainless steel insert weight of 58.5 g (Figure 1). After 2 days’ culture, the medium was removed and replaced with 10% formalin for 20 minutes, after which the weight, fabric mesh and coverslip were removed, and the gel left in formalin for a further 20 minutes. Gels were then washed twice in PBS and processed for Oil-Red-O staining as with the monolayers. The proportion of Oil-Red-O positive cells was evaluated using a 40x objective on a Leica DMIL microscope, on at least 100 cells from random fields for each condition. Cells with more than a couple of lipid droplets clearly within the cytoplasm were counted as positive. The scoring was done blind on the actual samples directly under the microscope, and reproduced by two independent experimentators. Each experiment was done in triplicate and, unless otherwise noted, a minimum of 3 experiments were performed for each condition. For the testing of the inhibitors, 10 µM Imatinib, 10 µM PP2 or 5 µg/ml 1H7 (with 10 µg/ml in the gel) were added to the medium immediately after gel polymerization (day 0) and maintained throughout.

### Quantitative Real-Time PCR

Gels were placed directly into TRIzol Reagent (Invitrogen) at 4°C for 1hr. Homogenization and phase separation were carried out according to the manufacturer’s instructions. The aqueous phase was harvested and used for RNA isolation using the RNeasy Mini Kit according to the standard protocol (Qiagen). Reverse transcription was carried out using the QuantiTect Reverse Transcription Kit (Qiagen) according to manufacturer’s instructions. PPARg gene expression was measured by q-PCR using the SYBR Green JumpStart Taq ReadyMix for quantitative PCR (Sigma-Aldrich, UK) with validated primers. The GAPDH gene was used as an endogenous control to normalize sample concentration, and all samples were run as 6 repeats. RT-PCR reactions were performed on an HT7900 Fast Real-Time PCR system (Applied Biosystems), and the final analysis was performed using DART-PCR v1.0 software [18]; the software summarises the Ct values and calculates the efficiency of amplification for each data set, in order to generate a relative expression value (R_0_) that can be compared across the samples. The R_0_ values of the gene of interest are normalised to that of the housekeeping gene for each sample in order to obtain, across the samples, the “fold” change in the gene of interest. The 6 replicates for each condition allow for internal variation and therefore permits any differences to be statistically significant.

### FACS Analysis and Thy-1 Population Sorting

Subconfluent (70–80% confluency) cells were trypsinised, washed, and a minimum of 300,000 cells per condition were transferred into 15 ml tubes. Cells were centrifuged and the pellet re-suspended in 500 µl of either PBS or PBS with primary antibody (1∶20 Anti-Thy-1 clone F15-42-1, Millipore). Cells were kept on ice for 1 hour with occasional mixing of the tube. Cells were then washed once in PBS, and further incubated with secondary antibody (1∶50 Alexa fluor 488 Affinipure donkey anti-mouse IgG (H+L), Stratech) in PBS for 1 hour on ice with occasional shaking. Cells were washed twice with PBS, re-suspended in 500 µl of PBS, transferred to a FACS tube (BD Falcon polystyrene 352052) and analysed on a FACSCalibur (Becton Dickinson). Between 1 and 2 x10^4^ events were counted for each sample, and the Geometric Mean fluorescent intensity (GMFI) evaluated after subtraction of the fraction of positive cells from the secondary antibody alone. As both control and GO cells had a dominant proportion of Thy-1-positive cells (albeit differing in the level of expression), the fraction of cells with low levels of expression of Thy-1 (fluorescence levels up to 10^3^) was chosen to calculate the percentage of Thy-1-low expressing cells. The HO2 GO cell line was chosen to separate Thy-1(−) and Thy-1(+) populations, as it displayed a significant proportion of both populations. HO2 cells were prepared for FACS analysis as described above and sorted on a Moflo XDP (Beckman Coulter, California) at the Institute of Child Health Cytometry Core Facility (UCL, London). The Alexa Fluor 488 signal was collected in FL1 channel through a 530/40 bandpass filter. A light scatter gate was drawn in the FSC *versus* SSC plot to exclude debris and clumps and include viable cells. Cells in this gate were displayed in a SSC *versus* SSC-W to further target single cells. Single and viable cells were then analysed in a SSC *versus* FL1 plot and a final gate was drawn to collect the labelled cells. A control sample (sham-treated HO2 cells, HO2s) was processed in an identical manner (including the journey to the cytometry facility) but not run through the FACS. Following sorting, the cells were plated in tissue culture dishes, allowed to recover for 24 hours, and processed for contraction or adipogenesis analyses within one passage.

### Statistical Analysis

All graphs show mean and SEM for at least 3 individual experiments (unless otherwise noted). Statistical analysis was performed using Student’s t test.

## Results

### GO Fibroblasts Display Increased Matrix Contraction Capability

**Figure 2:**
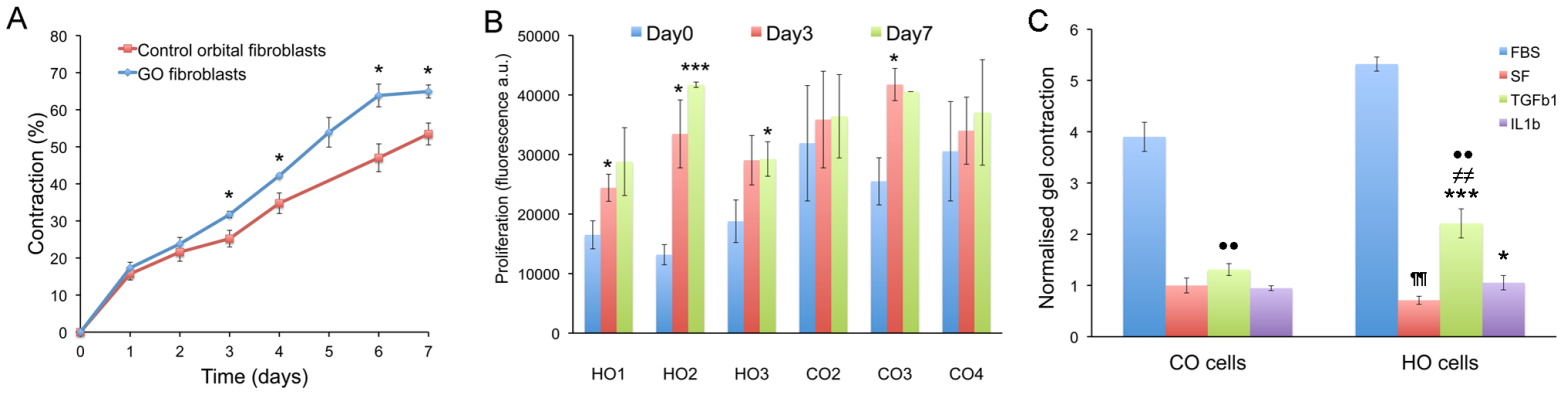
GO cells contract collagen matrices to a greater extent compared to control cells. (A) Control (red) and GO (blue) cells were embedded in free-floating collagen matrices in medium supplemented with 10% serum and contraction was monitored daily for 7 days. Contraction is expressed as the percentage of decrease in the gel area relative to the area at time 0. Shown is mean +/− SEM for 3 GO (HO1-3) and 3 control (CO2-4) fibroblast lines with 3–6 experiments in triplicate for each cell line. * Significant difference between GO and control cells, P<0.05. (B) Cell proliferation during gel contraction as measured using an Alamar Blue assay. Shown is mean +/− SEM each with 3 experiments in triplicate for each. *P<0.05, ***P<0.001, significant difference compared to level at day 0. (C) Day6 collagen gel contraction level in response to TGFβ1 and IL1β stimulation in all CO and HO fibroblasts (all normalized to the average Serum Free (SF) sample value for CO cells, n = 3 experiments, mean ± SEM). Statistical significance between SF and TGFβ1 or IL1β treated samples (*p<0.05, ***p<0.001) in each category; between SF in CO and HO cells (^¶¶^ P<0.01), between TGFβ1 treated samples of CO and HO cells (^≠≠^p<0.01), and TGFβ1 and IL1β treated samples between CO and HO cells respectively ( ^••^p<0.01).

Orbital fibrosis is a major pathological feature of GO. We have previously shown that fibroblasts isolated from pro-fibrotic periocular tissues display increased contractile properties as compared to normal counterparts [17]. We therefore hypothesised that the clinically-observed fibro-proliferative nature of GO could be reproduced using our standard 3D collagen gel contraction assay [17] [19], [20]. Although there was significant variation in each set (Figure S3A), GO orbital fibroblasts overall contracted collagen matrices more efficiently than control fibroblasts (Figure 2A). An Alamar Blue proliferation assay revealed that this increased contractility may be partly due to a higher proliferation rate for the GO cells in the gels (Figure 2B), although the proliferation levels were minimal, with no more than a 1.5 times increase at day 3 and 7 for all cell lines but HO2. In accordance with our previous work [19], there was overall no correlation between the cell proliferation rate and their ability to contract the matrix (Figure S3B). Incidentally, as increased fluorescence in the Alamar Blue assay signifies higher reduction levels in the environment, the lower baseline fluorescence levels observed at day 0 for the GO cells suggest that they intrinsically generate a more oxidative environment than the control cells, in agreement with a previously suggested link between GO and oxidative stress[21]–[23]. A range of inflammatory cytokines are known to promote fibroblasts contractile activity, with specific cytokines such as TGFβ1 linked to tissue scarring and fibrosis. To assess whether the increased contraction potential observed in GO cells following serum stimulation was linked to a greater sensitivity to inflammatory cytokines, we performed fibroblast-mediated gel contraction assays following stimulation with TGFβ1 or IL1β, two of the main cytokines linked to scarring and fibrosis. While control orbital fibroblasts responded only and poorly to TGFβ1, GO cells showed an increase in gel contraction following both IL1β and TGFβ1 stimulation, although the response to TGFβ1 was much stronger (Figure 2C). Neither control, nor GO cells proliferated in the assay (data not shown). Overall, this data suggested that the GO fibroblasts display an intrinsic fibro-proliferative phenotype, consistent with the disease pathology.

### GO Fibroblasts Undergo Spontaneous and Pressure-induced Adipogenesis in 3D Cultures

**Figure 3:**
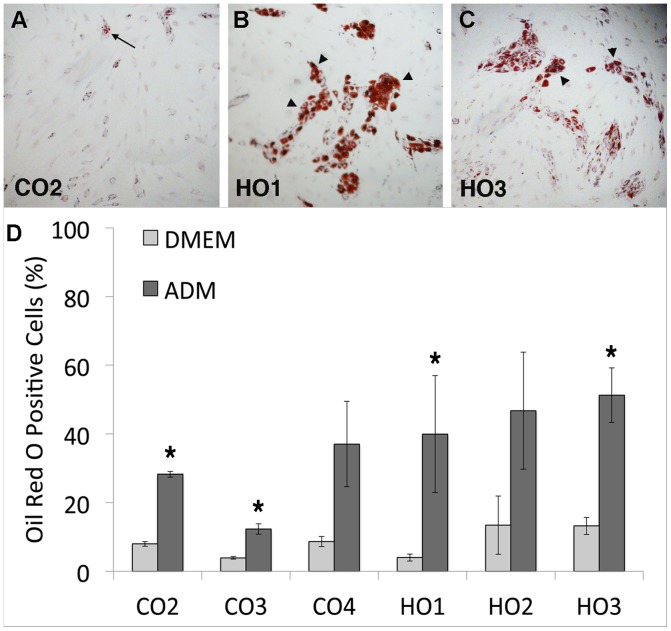
GO cells differentiate into adipocytes in monolayer cultures. (A–C) Representative images of orbital fibroblasts (A, Control-CO2; B, GO-HO1; C, GO- HO3) stained with Oil Red O after 2 weeks culture in adipocyte differentiation medium. Only a small proportion of cells containing a few lipid droplets were found in CO cells (arrow), while large clumps of highly positive cells (arrowheads) were present in HO cells. (D) Adipocyte differentiation in control and GO cells. Graph shows the percent of Oil-Red-O positive cells for each cell type (mean +/− SEM, n = 3–4). DMEM, standard culture medium; ADM, adipocyte differentiation medium. GO cells overall display significantly enhanced adipogenesis in presence of differentiation medium compared to control orbital fibroblasts (P = 0.03), and all cell lines show a significant increase in adipogenesis in ADM compared to regular DMEM (* p<0.05), with the exception of CO4 and HO2.

**Figure 4:**
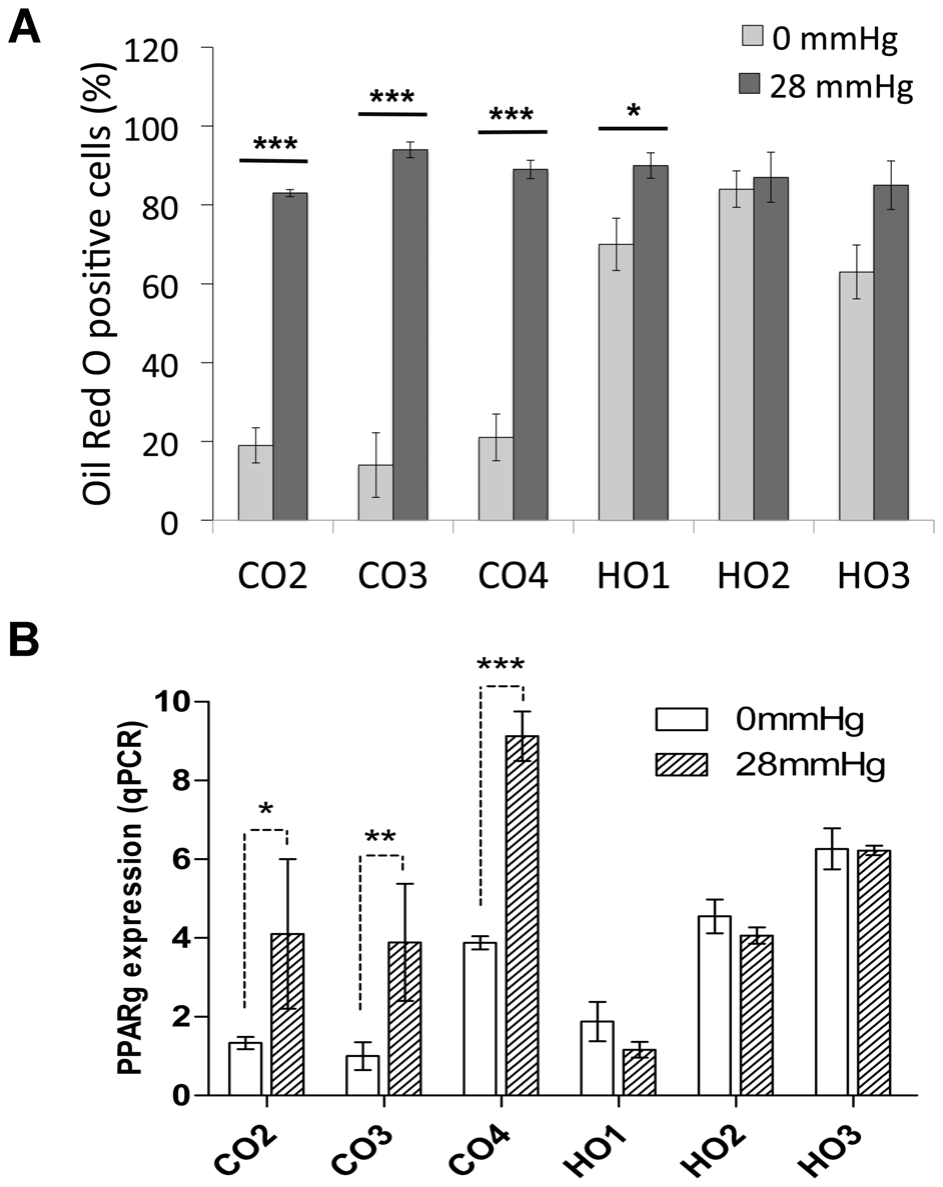
GO cells spontaneously differentiate into adipocytes in 3D cultures. Control **(**CO2-4) or GO (HO1-3) cells were seeded into collagen gels and cultured for 5 days in standard culture medium. The gels were then left untreated (0 mmHg) or subjected to pressure (28 mmHg) for a further 48 hours, followed by (A) fixation and staining with Oil-Red-O or (B) RNA extraction and qPCR. (A) Adipocyte differentiation in control and GO cells, shown as the percent of Oil-Red-O positive cells in each cell line (mean +/− SEM, n = 3). Student’s t test was used to compare adipogenesis levels between no pressure and pressure for each cell line (* p<0.05, ***p<0.001), and between control cells and GO cells overall (significantly different under no pressure condition, p = 0.001). (B) PPARg expression (normalised to GAPDH, levels) as measured by q-PCR. Shown is the mean +/− SEM for 3 experiments (* p<0.05, ** p<0.01, ***p<0.001).

Another key feature of GO is adipogenesis, where orbital fibroblasts trans-differentiate into adipocytes [5], [6]. To explore their adipogenic potential, we first determined the cells’ ability to differentiate into adipocytes in 2D monolayers using standard biochemical stimulation with a commercially available differentiation medium. As expected [10], even after 2 weeks, control orbital fibroblasts showed only a limited ability to differentiate into adipocytes - with a maximum of 37% cells (CO4) mildly positive for Oil-Red-O (Figure 3, A and D). By contrast, all three GO cell lines had, on average, more than 40% cells positive for Oil-Red-O, with most of the positive cells displaying a strong accumulation of lipid droplets (Figure 3, B–D). To better simulate the pathophysiological environment of orbital tissue in the context of active GO, fibroblasts were embedded in 3D collagen gels that were maintained attached within the wells for 5 days, so as to allow the cells to form a tissue-like environment and attain tensional homeostasis [24]. Custom weights were then placed onto the gels to increase pressure to the pathological levels measured in patients with active GO [13] and, after 48 hours, the gels were fixed and stained with Oil-Red-O or underwent RNA extraction for qPCR analysis. No significant changes in cell viability or morphology were identified after the application of pressure, with only a minor increase in the proportion of dead cells (Figure S4). Unexpectedly, after 7 days in 3D cultures, most GO fibroblasts spontaneously differentiated into adipocytes - with at least 60% of the cells presenting lipid droplets (Figure 4A) and expressing PPARg (Figure 4B). By contrast, control orbital cells displayed only minimal differentiation (20% or less Oil-Red-O positive cells and low PPARg expression levels). Most notably, CO4 cells showed elevated PPARg levels, matching the significant adipogenesis level measured in 2D after biochemical differentiation. However, all cell lines – both GO and control fibroblasts - showed maximum levels of adipogenesis (both Oil-Red-O staining and PPARg expression) when cultured under pressure (Figure 4, A and B), with the controls reaching similar levels as GO cells. This suggests that, although GO orbital fibroblasts clearly have an intrinsic pro-adipogenic phenotype, pressure applied to normal orbital fibroblasts is sufficient to induce trans-differentiation into adipocytes.

### A Thy-1-low Population in GO Cells Underlies the Adipogenic Phenotype

**Figure 5:**
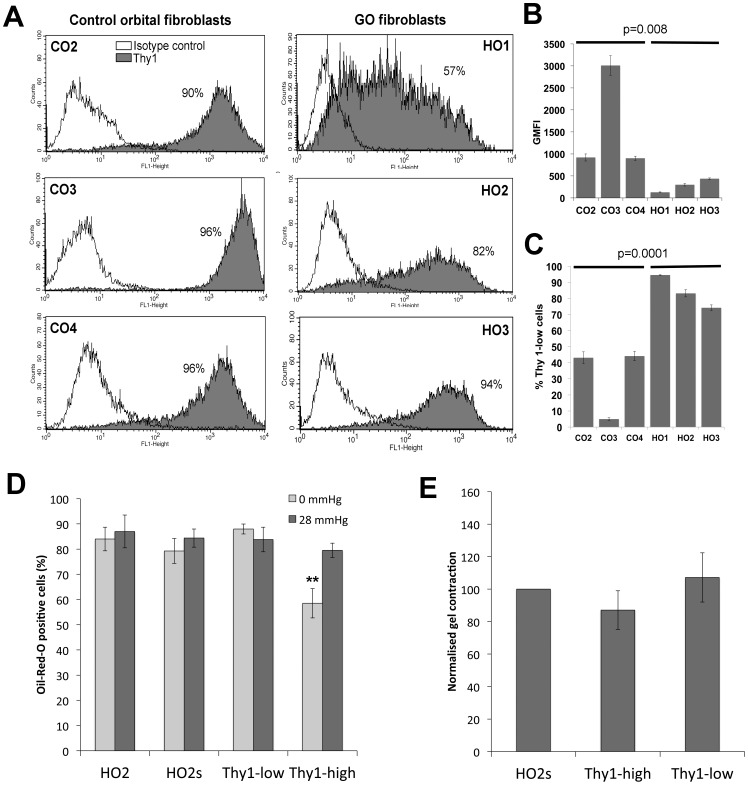
A Thy-1-low cell population in GO cells underlies the adipogenic, but not the contractile phenotype. (A) Expression levels of Thy-1 (CD90) protein were determined in control (CO2-4) and GO (HO1-3) cells by FACS analysis. Shown is a representative distribution for each cell line (from a minimum of 3 experiments), where white shows the baseline control distribution (IgG isotype control antibody), and grey depicts the Thy-1 expression profile (% Thy1 positive cells as indicated). (B) The Geometric Mean Fluorescent Intensity (GMFI) and (C) the proportion of Thy 1-low cells (fluorescence <1000) are represented as mean +/− SEM, from three independent experiments for each cell line. Statistical analysis using a Student’s t test shows a significant difference between control and GO fibroblasts for both parameters (p as indicated). (D, E) HO2 cells were FACS-sorted into Thy-1 negative/low expressor (Thy-1-low) and Thy-1 strongly positive (Thy-1-high) populations, or sham sorted (HO2s), and tested for their ability to differentiate into adipocyte in 3D (D), and to contract collagen matrices (E), immediately and/or within 1 passage after sorting. (D) Adipocyte differentiation: graph shows the percent of Oil-Red-O positive cells for each cell type (mean +/− SEM; HO2 and Thy-1-high n = 3, HO2s and Thy-1-low, 1 experiment in triplicates). ** significant difference between HO2 and Thy-1-high (p = 0.01). (E) Thy-1-low and Thy-1-high cells collagen matrix contraction at day7, relative to sham-sorted HO2 cells (mean +/− SEM for at least 3 experiments in triplicate for each cell line, no significant difference between the control and sorted cells).

As Thy-1 expression has previously been shown to define myofibroblastic or adipogenic phenotypes in myometrial and orbital fibroblasts [10], we hypothesized that its expression might be linked to the contractile and/or adipogenic phenotype of GO cells. FACS analysis revealed that control orbital fibroblasts expressed high levels of Thy-1, with a small fraction (if any) of negative- to low-expressors (Figure 5A). By contrast, GO cells presented significantly lower levels of Thy-1 expression, with a much lower level of geometric mean fluorescence intensity (GMFI), and a more spread overall expression pattern (Figure 5, A and B). While the control cells typically had fluorescence levels above 10^3^, most GO cells were much below this value (Figure 5 C).

To determine whether a Thy-1-low population in GO was linked to the observed contractile and adipogenic phenotypes, we attempted to isolate Thy-1-negative and Thy-1-positive cell populations to analyse their behaviour separately. We selected the HO2 GO cell line for this study, as it had the strongest contractile and adipogenic profiles, as well as a significant number of both Thy-1-low and Thy-1-high expressing cells. The HO2 cell line was initially sorted into fully separated Thy-1(−) and Thy-1(+) populations - comprising cells completely negative or expressing very low levels of Thy-1 (fluorescence levels 10^1^ to 3×10^2^) and cells expressing Thy-1 at around the peak of fluorescence (fluorescence levels 10^3^ to maximum), respectively. However, we found that these sorted populations were extremely unstable and both reverted to the original mixed phenotype within 1 passage or less. In an attempt to reduce the instability of the sorted cells, we adopted less stringent fluorescence selection criteria, defining a Thy-1-low and a Thy-1-high cell population respectively, comprising the cells from negative to about one-third of the maximum fluorescence (up to 2×10^3^; Thy-1-low) or all the cells above two-thirds of the peak fluorescence (fluorescence levels 3×10^3^ to maximum; Thy-1-high). The populations sorted in this way were more stable and did not revert back to the original HO2 mixed phenotype until passage 2 or later. Thy-1-low, Thy-1-high and a sham-treated HO2 line (labelled but not sorted) were examined for their contractile and adipogenic behaviour immediately after, or within 1 passage of sorting. While Thy-1-low cells had a high adipogenic profile similar to that of sham-sorted parental HO2s cells, Thy-1-high cells were significantly less adipogenic under unpressurized conditions (Figure 5D), this implying (as predicted) that the presence of a Thy-1-low population in GO cells might at least partly underlie their adipogenic phenotype. By contrast, when tested for gel contraction potential, neither the Thy-1-low, nor the Thy-1-high populations were significantly different from the HO2s cells (Figure 5E) - this suggesting that the contractile phenotype is not linked to Thy-1 expression.

### The Contractile and Adipogenic Phenotypes are Regulated by Separate Pathways

To further explore the dichotomy between the contractile and adipogenic phenotypes in GO, we tested three drugs broadly targeting pathways that could potentially regulate these processes. Imatinib, a tyrosine kinase inhibitor that targets the Bcr-Abl pathway as well as c-kit and PDGF-R, has been studied extensively as a candidate for reducing tissue remodelling in GO, through a blockade of the PDGF receptor [25], [26]. The broad spectrum Src family kinase (SFK) inhibitor PP2 was used to target SFKs, as SFKs have been implicated in adipogenesis [27], [28] and their expression is reduced in Thy-1 positive cells [29], [30]. PP2 was confirmed to block serum-mediated Src phosphorylation in GO cells (Figure S5A). Finally, we used the commercially available 1H7 antibody against the IGF-1 receptor, as the IGF-1 receptor is over-expressed in GO fibroblasts [31], [32] and data, including our own (Figure S5B), suggests that this antibody can reverse some of the clinical features of GO such as hyaluronan production [9], [33].

**Figure 6:**
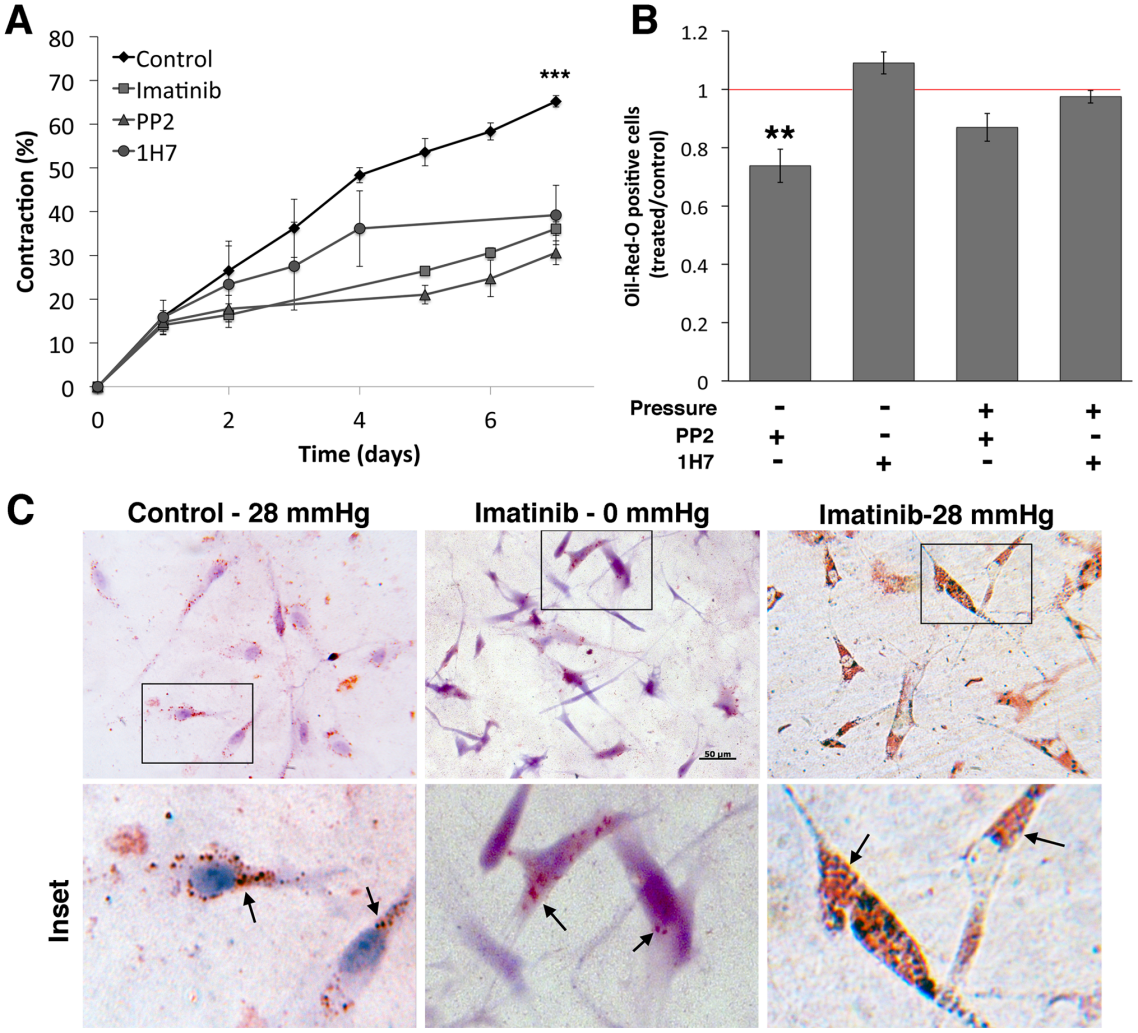
The contractile and adipogenic phenotypes are regulated by separate pathways. HO2 GO fibroblasts were embedded in collagen gels for either standard contraction assay (A) or 3D adipogenesis (B, C) in the presence of Imatinib (10 µM), PP2 (10 µM) or 1H7 (10 µg/ml in gel; 5 µg/ml in medium) inhibitors. (A) Collagen gel contraction in the presence of inhibitors (mean +/− SEM, for 3 experiments, each in triplicate). *** significant difference between control and treated samples, p<0.001. (B) Effect of inhibitor treatment on spontaneous adipogenesis in 3D, with/without pressure. Graph shows the frequency of Oil-Red-O positive cells in treated samples as ratioed to the proportion of positive cells in matching untreated control samples (mean +/− SEM, for 3 experiments, each in triplicate). ** significant difference between control and treated samples, p = 0.03. (C) Representative Oil-Red-O staining of HO2 cells in 3D gels, showing spontaneous adipogenesis upon pressure application (Control 28 mmHg). Treatment with Imatinib resulted in a further increase in the amount of lipid droplets in virtually all cells whether the samples were unpressured (Imatinib 0 mmHg) or under pressure (Imatinib 28 mmHg).

All 3 inhibitors significantly reduced gel contraction, by about 50% at day 7 (Figure 6A). However, 1H7 had no effect on adipogenesis (Figure 6B) - either with or without pressure - and Imatinib actually increased adipogenesis, with all treated cells displaying a massive accumulation of Oil-Red-O stained droplets (Figure 6C). In contrast, PP2 treatment significantly inhibited spontaneous adipogenesis in 3D, and also showed a tendency to reduce the pressure-induced adipogenesis (although the latter was not significant; Figure 6B). Overall, this suggested that, whilst contraction and adipogenesis are clearly regulated in part by separate pathways, both pathways may be modulated through common upstream SFK signalling.

## Discussion

One characteristic feature of GO is the occurrence of both adipogenesis and fibrosis as simultaneous pathological processes. The ability of GO fibroblasts to differentiate into adipocytes has been widely described [3], [5], [6], [10], [34], but little is known about their fibrotic potential. Using our well-characterised 3D model of collagen contraction, we have previously shown that fibroblasts derived from fibrotic ocular tissues display an increased contractile phenotype as compared to controls, and these fibroblasts retain, *in vitro*, both altered biomechanical properties and a specific molecular signature that might underlie their behaviour in disease [17]. Here we have uncovered a similar feature for orbital fibroblasts from patients with active Graves’ orbitopathy, suggesting that these cells have acquired an intrinsic fibro-proliferative phenotype and increased sensitivity to inflammatory cytokines. Whilst standard chemical stimulation of cellular monolayers confirmed the greater adipogenic potential of GO fibroblasts as compared to controls cells, we have shown that, unexpectedly, active GO fibroblasts spontaneously undergo adipogenic differentiation, without the need for any chemical stimulation, when placed in a pseudo-physiological 3D environment [24], [35], [36], suggesting that these cells are intrinsically primed for adipogenesis. We further demonstrated, using our pressure model, that healthy orbital fibroblasts - which normally do not spontaneously differentiate into adipocytes - can be induced to display a full adipocyte phenotype as a result of pressure stimulus alone. This suggests that the pathological intraorbital pressures encountered in GO [13] could be driving adipogenesis, potentially explaining clinical results suggesting that orbital decompression can contribute to a decrease in disease activity [37].

Although both fibrosis [38] and adipogenesis [16], [39] are influenced by mechanical cues, these two processes are usually mutually exclusive [16]. To explain the unusual co-occurrence of these two processes in the Graves’ orbit, we postulated that orbital fibroblasts might comprise a mixed cell population and further investigated the expression of Thy-1 as a marker that might differentiate the phenotypes [6]. In accordance with previous findings [6], [40], we identified a subpopulation of Thy-1-low expressors in our GO fibroblast lines, and showed that this population likely underlies the adipogenic phenotype of the GO cell lines. Interestingly, strict separation of the Thy-1 positive and the Thy-1 negative populations rendered them very unstable - reverting back to their original mixed phenotype within one passage. A previous study used multiple rounds of magnetic bead separation to successfully isolate the Thy-1(+) and Thy-1(−) populations, which might have influenced the stability of their phenotype [40]. Alternatively, the instability might be related to the sample origin, our tissues being derived from patients with extremely severe and active Graves’ orbitopathy. Sample origin might also explain the contradictory findings in another study where - using samples from patients with less severe disease - the authors showed enhanced Thy-1 expression in GO fibroblasts, rather than a decreased expression [5]. Despite Thy-1 expression having previously been linked to the contractile phenotype [29], [38], we could not show any difference in contractile efficiency between Thy-1-low or Thy-1-high cells - indicating that, while Thy-1 expression might govern adipogenic differentiation, it has no impact on the contractile phenotype.

Using 3 drugs previously reported as targeting pathways with therapeutic potential in GO, we showed that our 3D gel model could be used as an *in-vitro* drug assay for contraction and adipogenesis in GO. Significant evidence has implicated IGF1-R in the pathogenesis of GO [32], [41] and IGF-1 induces hyaluronan synthesis in GO fibroblasts [9], as well as cytokine responses [42], [43]. Identification of the co-localisation of the receptors for TSH and IGF-1 further supports the targeting of IGF-1 signalling as a potential treatment for GO [44], and a multicentre trial of an IGF-1R inhibitor in GO is currently recruiting [45]. We found no effect of IGF-1R inhibition on adipogenesis, but significant attenuation of the contractile phenotype - suggesting that IGF-1R antagonists might target the proliferative/fibrotic component of GO. PDGF inhibitors, including Imatinib, have also been suggested as a possible treatment, as inhibition of this pathway also reduce hyaluronan synthesis [25], [26], [46]. We show that, whilst PDGF-R inhibition was effective at reducing contraction, it resulted in an unanticipated and exaggerated adipogenic response in GO fibroblasts - as previously reported [47]. These untoward effects might limit further exploration of Imatinib as a treatment for GO. Although SFKs have not so far been directly linked to GO, SFKs have been linked to both adipogenesis and Thy-1 expression [27]–[30]. We show here that a broad spectrum SFK inhibitor alters both the contractile and the adipogenic potential of GO fibroblasts. Interestingly, SFK inhibition also shows a tendency towards reducing pressure-induced adipogenesis, which might indicate an effect on some components of the biomechanical pathway. Overall these results show that the adipogenic and the contractile phenotype of GO cells are – at least in part - mediated through separate pathways as some inhibitors affect only one of the two processes, but that they both may be regulated through a common upstream pathway involving SFKs.

Although significant progress has been made in developing animal models for GO [48], [49], these have still not been shown to have reproducible validity. The development of functional *in vitro* test models for GO might, therefore, provide an economical alternative on the translational pathway from basic science to therapeutic interventions. We have demonstrated that GO orbital fibroblasts display an intrinsic dual adipogenic/fibrotic phenotype, and marked sensitivity to the mechanical environment in a three-dimensional matrix. The novel three-dimensional cell culture provides an environment of more physiological relevance, this facilitating further exploration of the mechanisms and pathogenesis of GO – a disease fundamentally associated with gross disturbance of the mechanical micro-environment.

## Supporting Information

Figure S1 **Control and GO orbital fibroblasts express classical orbital fat fibroblast markers.** Immunocytochemistry for vimentin, cytokeratin and Factor VIII was performed on cytospins of control (CO2-4) and GO (HO1-3) cells using standard methods, and the slides were counterstained with H&E staining. Both sets of orbital fibroblasts were positive for mesanchymal cell marker vimentin, largely negative for epithelial marker cytokeratin and fully negative for endothelial marker Factor VIII.
(TIF)

Figure S2 **Control and GO orbital fibroblasts do not express CD45 fibrocyte marker.** FACS analysis was performed on control (CO2-4) and GO (HO1-3) for the fibrocyte marker CD45, with all cell lines showing a complete absence of staining for the marker.
(TIF)

Figure S3 **Orbital fibroblast matrix contraction potential is not correlated to cell proliferation in the gels.** (A) Individual contraction curves for control (CO2-4, red) and GO (HO1-3, blue) fibroblasts in the standard collagen gel contraction assay. Each curve shows the mean +/− SEM for 3–6 individual experiments, each in triplicate. (B) Representation of the gel contraction at day 3 and day 7 as a function of the proliferation rate (normalised to the value at day 0) demonstrates an absence of correlation between the two parameters. Each point represents one individual cell line at day 3 (red) and 7 (green), with a minimum of 3 experiments for each. The linear trends at day 3 and day 7 are shown with corresponding R2 value. The overall correlation coefficient between gel contraction and proliferation for all data points (day3 and 7 together) is 0.35.
(TIF)

Figure S4 **Cell viability in attached gels under pressure.** Control CO4 and GO HO1 fibroblasts were seeded in attached collagen gels as per our standard 3D adipogenesis protocol with 0 or 28 mmHg applied at day 5, and a LIVE (green)/DEAD (red) cytotoxicity assay was performed at day 7. Only a minor proportion of the cells were dead after 7 days in the gels without pressure (0 mmHg), with no difference between control and GO cells. There was a small increase in the proportion of dead cells in the samples that were under pressure for 48 hrs (28 mmHg), although only mildly significant in control cells (P as indicated on graph). There was no significant difference in the proportion of dead cells in control and GO cells under pressure. Arrows on the images point to dead cells.
(TIF)

Figure S5 **Activity of PP2 and 1H7 inhibitors on GO cells.** (A) SFK inhibitor PP2 blocks serum-induced Src phosphorylation in GO fibroblasts. HO2 cells were starved ON and stimulated with 15% serum in the presence/absence of 20 uM PP2. Shown is a representative Western blot for phosphorylated Src at time 0, 5 and 30 min after serum stimulation. GAPDH was used as the loading control. (B) 1H7 anti-IGF-1R antibody blocks IGF-1 induced hyaluronan (HA) secretion by GO fibroblasts. HO1 GO cells were starved overnight in medium with 1% serum, and further incubated for 48 hrs in presence/absence of rIGF-1 (10 nM/L) with/without 1H7 antibody (5ug/ml). The amount of HA produced by the cells was measured by ELISA and normalised to cell numbers determined by Alamar Blue Assay. IGF-1 treatment results in a significant increase in HA production (P<0.001), which is inhibited by treatment with 1H7 (P<0.001). Shown is an average of 3 experiments, each in triplicate.
(TIF)

Methods S1 (DOCX)

## References

[1] Garrity JA, Bahn RS (2006) Pathogenesis of Graves Ophthalmopathy: Implications for Prediction, Prevention, and Treatment. Am J Ophthalmol 142: 147–153.e2 10.1016/j.ajo.2006.02.047 16815265 PMC3960010

[2] Sisson JC, Spaugh BI, Vanderburg JA (1970) Functional aspects of fibroblasts derived from the retrobulbar tissue of man. Exp Eye Res 10: 201–206. 4249556 10.1016/s0014-4835(70)80028-8

[3] Kumar S (2004) Evidence for Enhanced Adipogenesis in the Orbits of Patients with Graves’ Ophthalmopathy. Journal of Clinical Endocrinology & Metabolism 89: 930–935 10.1210/jc.2003031427 14764816 PMC3902012

[4] Zhang L, Grennan-Jones F, Lane C, Rees DA, Dayan CM, et al (2012) Adipose Tissue Depot-Specific Differences in the Regulation of Hyaluronan Production of Relevance to Graves’ Orbitopathy. J Clin Endocrinol Metab 97: 653–662 10.1210/jc.2011-1299 22162480

[5] Khoo TK, Coenen MJ (2008) Schiefer AR, Kumar S, Bahn RS (2008) Evidence for enhanced Thy-1 (CD90) expression in orbital fibroblasts of patients with Graves’ ophthalmopathy. Thyroid 18: 1291–1296 10.1089/thy.2008.0255 18976167 PMC2857447

[6] Smith TJ, Koumas L, Gagnon A, Bell A, Sempowski GD, et al (2001) Orbital fibroblast heterogeneity may determine the clinical presentation of thyroid-associated ophthalmopathy. J Clin Endocrinol Metab 87: 385–392. 10.1210/jcem.87.1.8164 11788681

[7] Smith TJ (2010) Potential role for bone marrow-derived fibrocytes in the orbital fibroblast heterogeneity associated with thyroid-associated ophthalmopathy. Clinical & Experimental Immunology 162: 24–31 10.1111/j.1365-2249.2010.04219.x 20659126 PMC2990926

[8] Hoa N, Tsui S, Afifiyan NF, Sinha Hikim A, Li B, et al (2012) Nuclear Targeting of IGF-1 Receptor in Orbital Fibroblasts from Graves’ Disease: Apparent Role of ADAM17. PLoS ONE 7: e34173 10.1371/journal.pone.0034173.g007 22506015 PMC3323600

[9] Smith TJ (2004) Immunoglobulins from Patients with Graves’ Disease Induce Hyaluronan Synthesis in Their Orbital Fibroblasts through the Self-Antigen, Insulin-Like Growth Factor-I Receptor. J Clin Endocrinol Metab 89: 5076–5080 10.1210/jc.2004-0716 15472208

[10] Koumas L, Smith TJ, Feldon S, Blumberg N, Phipps RP (2003) Thy-1 Expression in Human Fibroblast Subsets Defines Myofibroblastic or Lipofibroblastic Phenotypes. The American Journal of Pathology 163: 1291–1300 10.1016/S0002-9440(10)63488-8 14507638 PMC1868289

[11] Ezra DG, Beaconsfield M, Collin R (2009) Surgical anatomy of the upper eyelid: old controversies, new concepts. Ex Rev Op 4: 47–57 10.1586/17469899.4.1.47

[12] Riemann CD, Foster JA, Kosmorsky GS (1999) Direct orbital manometry in healthy patients. Ophthalmic Plastic and Reconstructive Surgery 15: 121–125. 10189640 10.1097/00002341-199903000-00010

[13] Berthout A, Vignal C, Jacomet PV, Galatoire O, Morax S (2010) Intraorbital pressure measured before, during, and after surgical decompression in Graves’ orbitopathy. J FR Ophthalmol 33: 623–629. 10.1016/j.jfo.2010.08.004 21047700

[14] Discher DE, Janmey P, Wang Y-L (2005) Tissue cells feel and respond to the stiffness of their substrate. Science 310: 1139–1143 10.1126/science.1116995 16293750

[15] Chen WLK, Simmons CA (2011) Lessons from (patho)physiological tissue stiffness and their implications for drug screening, drug delivery and regenerative medicine. Adv Drug Deliv Rev 63: 269–276 10.1016/j.addr.2011.01.004 21241759

[16] McBeath R, Pirone DM, Nelson CM, Bhadriraju K, Chen CS (2004) Cell shape, cytoskeletal tension, and RhoA regulate stem cell lineage commitment. Dev Cell 6: 483–495. 15068789 10.1016/s1534-5807(04)00075-9

[17] Ezra DG, Ellis JS, Beaconsfield M, Collin R, Bailly M (2010) Changes in fibroblast mechanostat set point and mechanosensitivity: an adaptive response to mechanical stress in floppy eyelid syndrome. Invest Ophthalmol Vis Sci 51: 3853–3863 10.1167/iovs.09-4724 20220050 PMC2910631

[18] Peirson SN (2003) Experimental validation of novel and conventional approaches to quantitative real-time PCR data analysis. Nucleic Acids Research 31: 73e–73 10.1093/nar/gng073 PMC167648 12853650

[19] Martin-Martin B, Tovell V, Dahlmann-Noor AH, Khaw PT, Bailly M (2011) The effect of MMP inhibitor GM6001 on early fibroblast-mediated collagen matrix contraction is correlated to a decrease in cell protrusive activity. European Journal of Cell Biology 90: 26–36 10.1016/j.ejcb.2010.09.008 21040999 PMC7611814

[20] Tovell VE, Chau CY, Khaw PT, Bailly M (2012) Rac1 Inhibition Prevents Tissue Contraction and MMP Mediated Matrix Remodeling in the Conjunctiva. Invest Ophthalmol Vis Sci 53: 4682–4691 10.1167/iovs.11-8577 22695959

[21] Bartalena L, Tanda ML, Piantanida E, Lai A (2003) Oxidative stress and Graves’ ophthalmopathy: in vitro studies and therapeutic implications. Biofactors 19: 155–163. 14757966 10.1002/biof.5520190308

[22] Hondur A, Konuk O, Dincel AS, Bilgihan A, Unal M, et al (2008) Oxidative stress and antioxidant activity in orbital fibroadipose tissue in Graves’ ophthalmopathy. Curr Eye Res 33: 421–427 10.1080/02713680802123532 18568878

[23] Zarković M (2012) The role of oxidative stress on the pathogenesis of graves’ disease. J Thyroid Res 2012: 302537 10.1155/2012/302537 22175033 PMC3235898

[24] McGee KM, Vartiainen MK, Khaw PT, Treisman R, Bailly M (2011) Nuclear transport of the serum response factor coactivator MRTF-A is downregulated at tensional homeostasis. EMBO Rep 12: 963–970 10.1038/embor.2011.141 21799516 PMC3166461

[25] van Steensel L, Paridaens D, Dingjan GM, van Daele PLA, van Hagen PM, et al (2010) Platelet-Derived Growth Factor-BB: A Stimulus for Cytokine Production by Orbital Fibroblasts in Graves’ Ophthalmopathy. Invest Ophthalmol Vis Sci 51: 1002–1007 10.1167/iovs.09-4338 19797221

[26] van Steensel L, Paridaens D, Schrijver B, Dingjan GM, van Daele PLA, et al (2009) Imatinib Mesylate and AMN107 Inhibit PDGF-Signaling in Orbital Fibroblasts: A Potential Treatment for Graves’ Ophthalmopathy. Invest Ophthalmol Vis Sci 50: 3091–3098 10.1167/iovs.08-2443 19234339

[27] Sun Y, Ma Y-C, Huang J, Chen KY, McGarrigle DK, et al (2005) Requirement of SRC-family tyrosine kinases in fat accumulation. Biochemistry 44: 14455–14462 10.1021/bi0509090 16262245

[28] Mastick CC, Saltiel AR (1997) Insulin-stimulated tyrosine phosphorylation of caveolin is specific for the differentiated adipocyte phenotype in 3T3-L1 cells. J Biol Chem 272: 20706–20714. 9252391 10.1074/jbc.272.33.20706

[29] Rege TA, Pallero MA, Gomez C, Grenett HE, Murphy-Ullrich JE, et al (2006) Thy-1, via its GPI anchor, modulates Src family kinase and focal adhesion kinase phosphorylation and subcellular localization, and fibroblast migration, in response to thrombospondin-1/hep I. Exp Cell Res. 312: 3752–3767 10.1016/j.yexcr.2006.07.029 17027000

[30] Pei Y, Sherry DM, McDermott AM (2004) Thy-1 distinguishes human corneal fibroblasts and myofibroblasts from keratocytes. Exp Eye Res 79: 705–712 10.1016/j.exer.2004.08.002 15500828

[31] Hoa N, Tsui S, Afifiyan NF, Sinha Hikim A, Li B, et al (2012) Nuclear targeting of IGF-1 receptor in orbital fibroblasts from Graves’ disease: apparent role of ADAM17. PLoS ONE 7: e34173 10.1371/journal.pone.0034173 22506015 PMC3323600

[32] Naik VM, Naik MN, Goldberg RA, Smith TJ, Douglas RS (2010) Immunopathogenesis of Thyroid Eye Disease: Emerging Paradigms. Survey of Ophthalmology 55: 215–226 10.1016/j.survophthal.2009.06.009 20385333 PMC2854657

[33] Kumar S, Iyer S, Bauer H, Coenen M, Bahn RS (2012) A stimulatory thyrotropin receptor antibody enhances hyaluronic acid synthesis in graves’ orbital fibroblasts: inhibition by an IGF-I receptor blocking antibody. J Clin Endocrinol Metab 97: 1681–1687 10.1210/jc.2011-2890 22399503 PMC3339886

[34] Crisp M, Starkey KJ, Lane C, Ham J, Ludgate M (2000) Adipogenesis in thyroid eye disease. Invest Ophthalmol Vis Sci 41: 3249–3255. 11006210

[35] Baker BM, Chen CS (2012) Deconstructing the third dimension - how 3D culture microenvironments alter cellular cues. Journal of cell science. 10.1242/jcs.079509 PMC3434846 22797912

[36] Cukierman E (2001) Taking Cell-Matrix Adhesions to the Third Dimension. Science 294: 1708–1712 10.1126/science.1064829 11721053

[37] Oh S-R, Tung JD, Priel A, Levi L, Granet DB, et al (2013) Reduction of Orbital Inflammation following Decompression for Thyroid-Related Orbitopathy. BioMed Research International 2013: 1–6 10.1016/j.ophtha.2005.10.060 PMC3703426 23853771

[38] Huang C, Ogawa R (2012) Fibroproliferative disorders and their mechanobiology. Connect Tissue Res 53: 187–196 10.3109/03008207.2011.642035 22329637

[39] Stacey DH, Hanson SE, Lahvis G, Gutowski KA, Masters KS (2009) In vitro adipogenic differentiation of preadipocytes varies with differentiation stimulus, culture dimensionality, and scaffold composition. Tissue Eng Part A 15: 3389–3399 10.1089/ten.TEA.2008.0293 19402786

[40] Koumas L, Smith TJ, Phipps RP (2002) Fibroblast subsets in the human orbit: Thy-1+ and Thy-1- subpopulations exhibit distinct phenotypes. Eur J Immunol 32: 477–485 doi:;10.1002/1521-4141(200202)32:2<477::AID-IMMU477>3.0.CO;2-U 11813166 10.1002/1521-4141(200202)32:2<477::AID-IMMU477>3.0.CO;2-U

[41] Hoa N, Tsui S, Afifiyan NF, Sinha Hikim A, Li B, et al (2012) Nuclear Targeting of IGF-1 Receptor in Orbital Fibroblasts from Graves’ Disease: Apparent Role of ADAM17. PLoS ONE 7: e34173 10.1371/journal.pone.0034173.g007 22506015 PMC3323600

[42] Smith TJ (2003) The putative role of fibroblasts in the pathogenesis of Graves’ disease: evidence for the involvement of the insulin-like growth factor-1 receptor in fibroblast activation. Autoimmunity 36: 409–415. 14669949 10.1080/08916930310001603000

[43] Pritchard J, Han R, Horst N, Cruikshank WW, Smith TJ (2003) Immunoglobulin activation of T cell chemoattractant expression in fibroblasts from patients with Graves’ disease is mediated through the insulin-like growth factor I receptor pathway. J Immunol 170: 6348–6354. 12794168 10.4049/jimmunol.170.12.6348

[44] Tsui S, Naik V, Hoa N, Hwang CJ, Afifiyan NF, et al (2008) Evidence for an association between thyroid-stimulating hormone and insulin-like growth factor 1 receptors: a tale of two antigens implicated in Graves’ disease. J Immunol 181: 4397–4405. 18768899 10.4049/jimmunol.181.6.4397 PMC2775538

[45] www.clinicaltrials.gov (n.d.) www.clinicaltrials.gov. Available: http://www.clinicaltrials.gov.Accessed 2013 Aug 4.

[46] Kang SM, Lee SY (2013) Effects of PDGF-BB and b-FGF on the production of cytokines, hyaluronic acid and the proliferation of orbital fibroblasts in thyroid ophthalmopathy. Mol Cell Toxicol 9: 195–202 10.1007/s13273-013-0024-1

[47] Borriello A, Caldarelli I, Basile MA, Bencivenga D, Tramontano A, et al (2011) The Tyrosine Kinase Inhibitor Dasatinib Induces a Marked Adipogenic Differentiation of Human Multipotent Mesenchymal Stromal Cells. PLoS ONE 6: e28555 10.1371/journal.pone.0028555 22164306 PMC3229607

[48] Zhao SX, Tsui S, Cheung A, Douglas RS, Smith TJ, et al (2011) Orbital fibrosis in a mouse model of Graves’ disease induced by genetic immunization of thyrotropin receptor cDNA. Journal of Endocrinology 210: 369–377 10.1530/JOE-11-0162 21715431 PMC3152291

[49] Moshkelgosha S, So P-W, Deasy N, Diaz-Cano S, Banga JP (2013) Cutting Edge: Retrobulbar Inflammation, Adipogenesis, and Acute Orbital Congestion in a Preclinical Female Mouse Model of Graves’ Orbitopathy Induced by Thyrotropin Receptor Plasmid-in Vivo Electroporation. Endocrinology 154: 3008–3015 10.1210/en.2013-1576 23900776

