# Supplementary figures and images for: Independent Adipogenic and Contractile Properties of Fibroblasts in Graves’ Orbitopathy: An In Vitro Model for the Evaluation of Treatments
Source: PLoS One. 2014 Apr 21;9(4):e95586. doi: 10.1371/journal.pone.0095586 (PMC3994071; doi:10.1371/journal.pone.0095586)

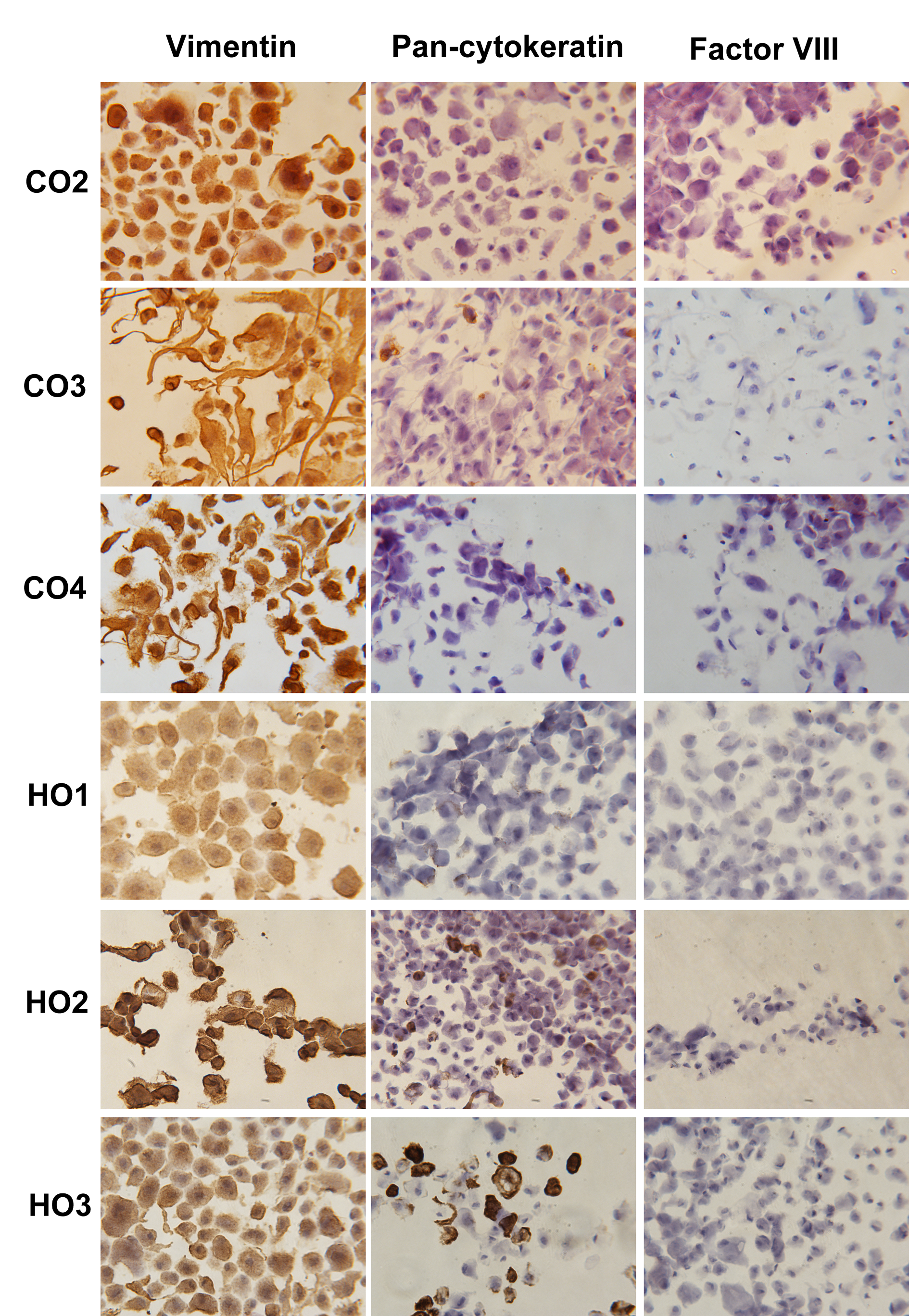

Supplement: Figure S1 — Control and GO orbital fibroblasts express classical orbital fat fibroblast markers. Immunocytochemistry for vimentin, cytokeratin and Factor VIII was performed on cytospins of control (CO2-4) and GO (HO1-3) cells using standard methods, and the slides were counterstained with H&E staining. Both sets of orbital fibroblasts were positive for mesanchymal cell marker vimentin, largely negative for epithelial marker cytokeratin and fully negative for endothelial marker Factor VIII. (TIF) [file pone.0095586.s001.tif]

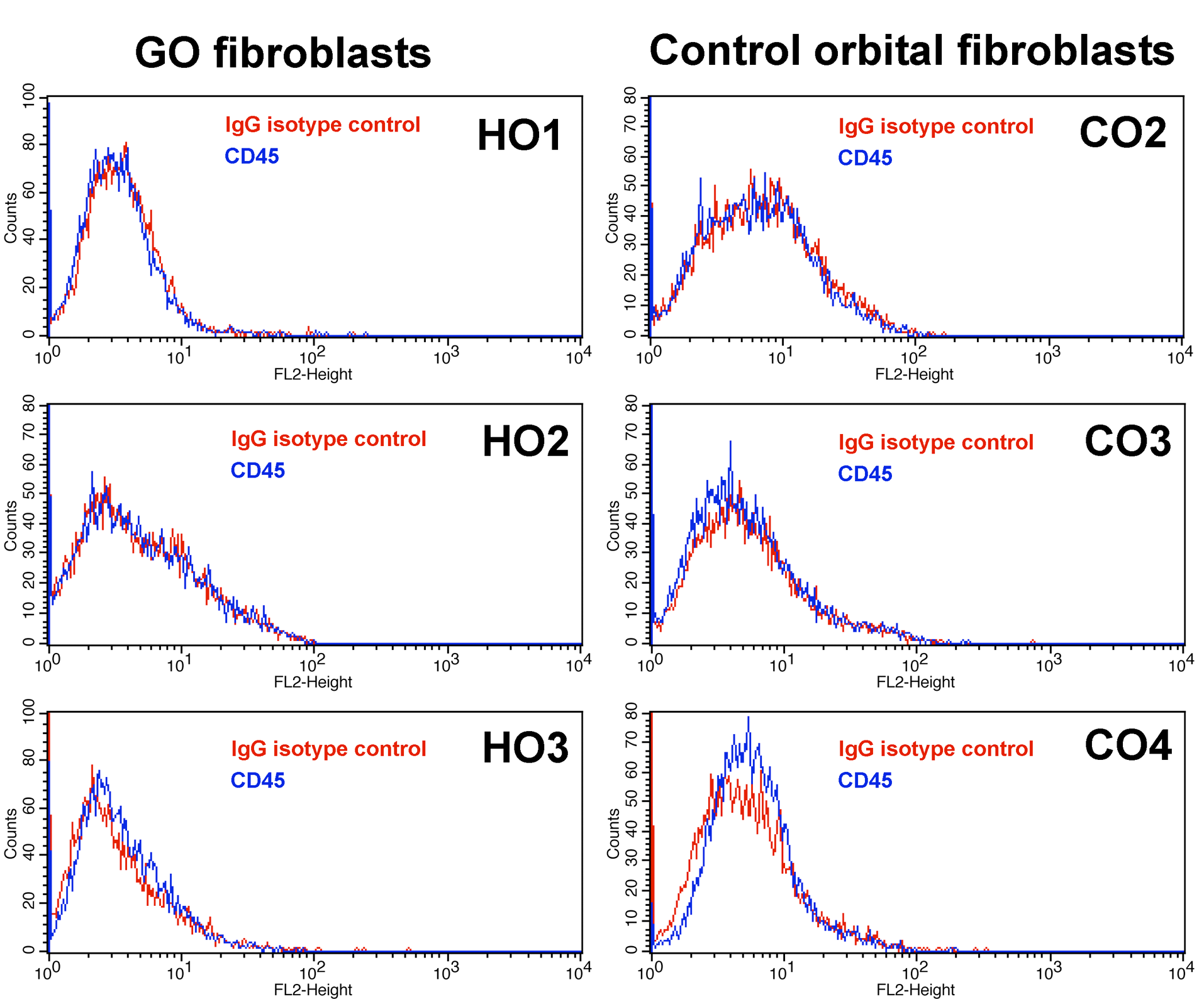

Supplement: Figure S2 — Control and GO orbital fibroblasts do not express CD45 fibrocyte marker. FACS analysis was performed on control (CO2-4) and GO (HO1-3) for the fibrocyte marker CD45, with all cell lines showing a complete absence of staining for the marker. (TIF) [file pone.0095586.s002.tif]

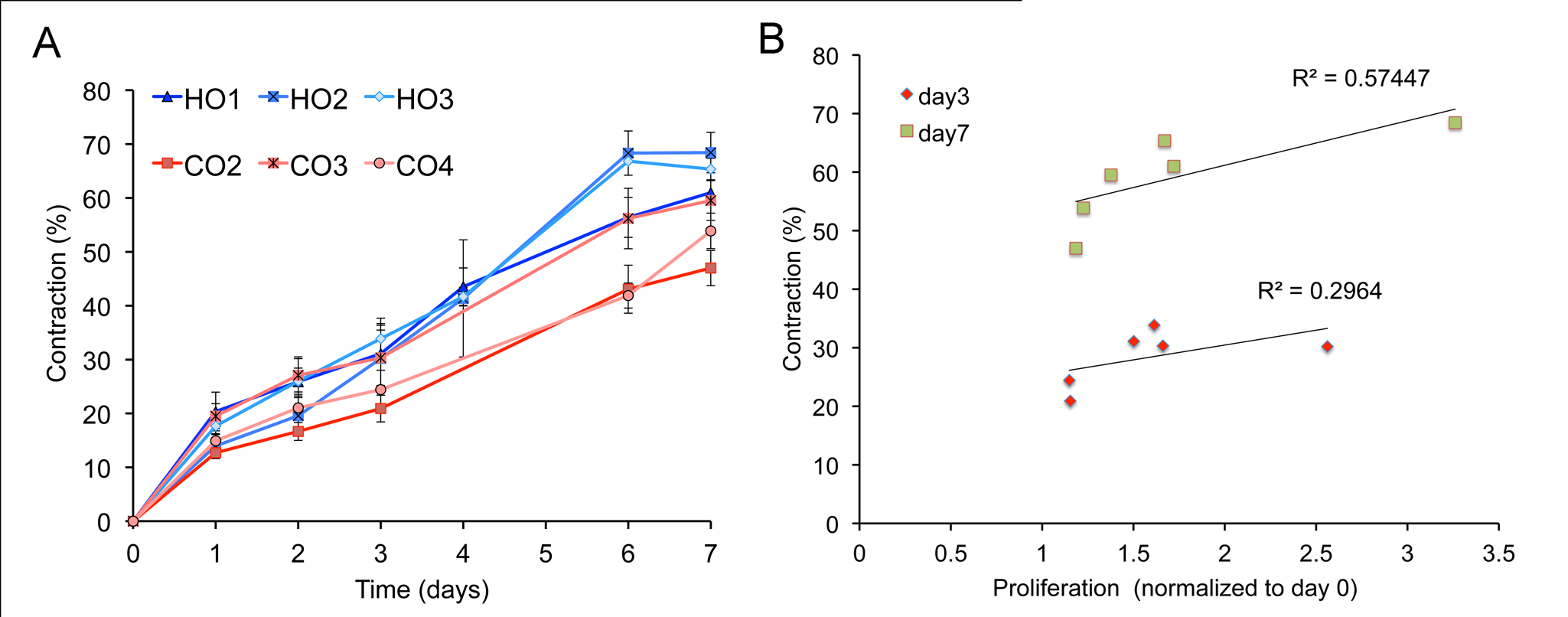

Supplement: Figure S3 — Orbital fibroblast matrix contraction potential is not correlated to cell proliferation in the gels. (A) Individual contraction curves for control (CO2-4, red) and GO (HO1-3, blue) fibroblasts in the standard collagen gel contraction assay. Each curve shows the mean +/− SEM for 3–6 individual experiments, each in triplicate. (B) Representation of the gel contraction at day 3 and day 7 as a function of the proliferation rate (normalised to the value at day 0) demonstrates an absence of correlation between the two parameters. Each point represents one individual cell line at day 3 (red) and 7 (green), with a minimum of 3 experiments for each. The linear trends at day 3 and day 7 are shown with corresponding R2 value. The overall correlation coefficient between gel contraction and proliferation for all data points (day3 and 7 together) is 0.35. (TIF) [file pone.0095586.s003.tif]

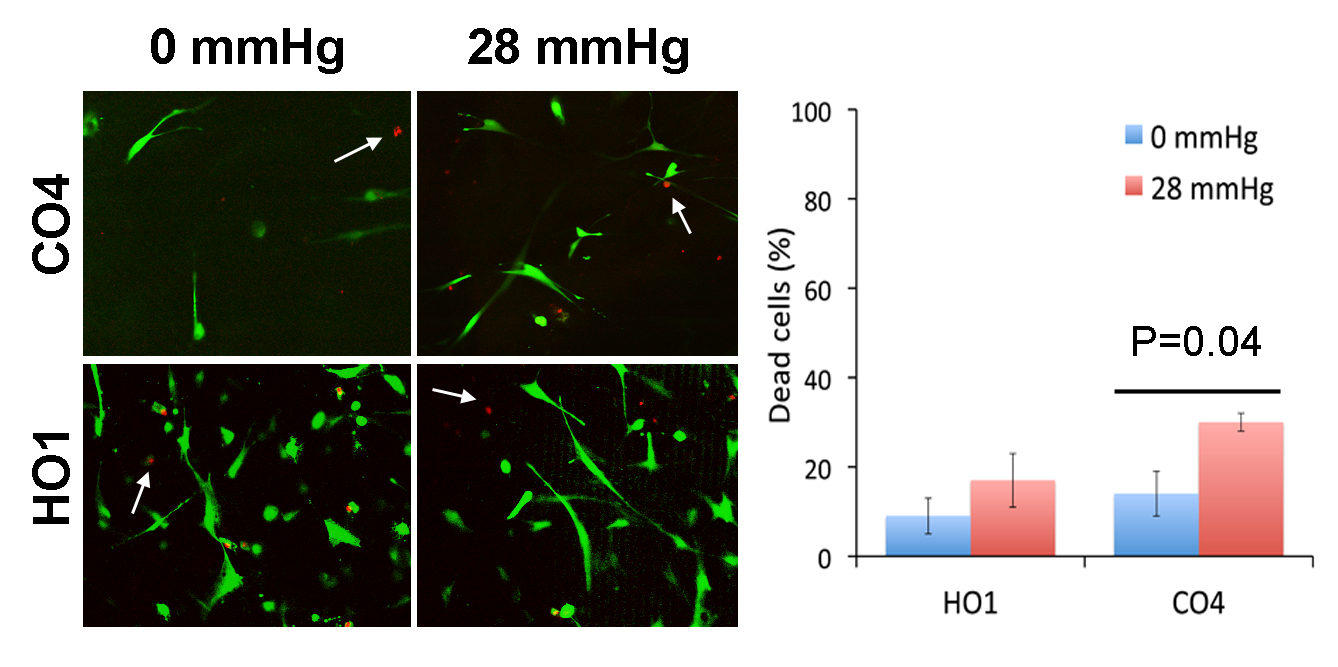

Supplement: Figure S4 — Cell viability in attached gels under pressure. Control CO4 and GO HO1 fibroblasts were seeded in attached collagen gels as per our standard 3D adipogenesis protocol with 0 or 28 mmHg applied at day 5, and a LIVE (green)/DEAD (red) cytotoxicity assay was performed at day 7. Only a minor proportion of the cells were dead after 7 days in the gels without pressure (0 mmHg), with no difference between control and GO cells. There was a small increase in the proportion of dead cells in the samples that were under pressure for 48 hrs (28 mmHg), although only mildly significant in control cells (P as indicated on graph). There was no significant difference in the proportion of dead cells in control and GO cells under pressure. Arrows on the images point to dead cells. (TIF) [file pone.0095586.s004.tif]

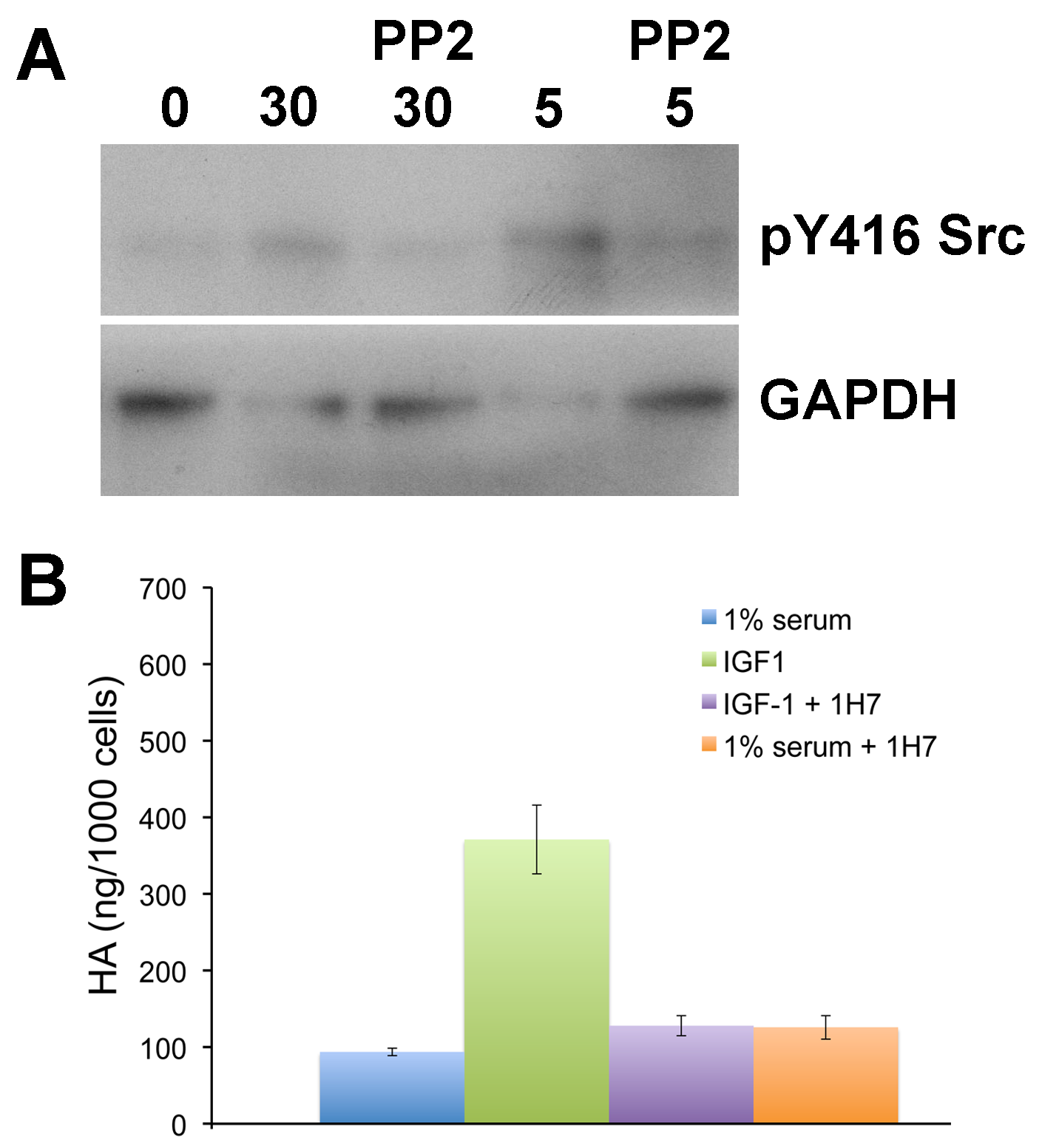

Supplement: Figure S5 — Activity of PP2 and 1H7 inhibitors on GO cells. (A) SFK inhibitor PP2 blocks serum-induced Src phosphorylation in GO fibroblasts. HO2 cells were starved ON and stimulated with 15% serum in the presence/absence of 20 uM PP2. Shown is a representative Western blot for phosphorylated Src at time 0, 5 and 30 min after serum stimulation. GAPDH was used as the loading control. (B) 1H7 anti-IGF-1R antibody blocks IGF-1 induced hyaluronan (HA) secretion by GO fibroblasts. HO1 GO cells were starved overnight in medium with 1% serum, and further incubated for 48 hrs in presence/absence of rIGF-1 (10 nM/L) with/without 1H7 antibody (5ug/ml). The amount of HA produced by the cells was measured by ELISA and normalised to cell numbers determined by Alamar Blue Assay. IGF-1 treatment results in a significant increase in HA production (P<0.001), which is inhibited by treatment with 1H7 (P<0.001). Shown is an average of 3 experiments, each in triplicate. (TIF) [file pone.0095586.s005.tif]
