# Supplementary material for: Independent Adipogenic and Contractile Properties of Fibroblasts in Graves’ Orbitopathy: An In Vitro Model for the Evaluation of Treatments
Source: PLoS One. 2014 Apr 21;9(4):e95586. doi: 10.1371/journal.pone.0095586 (PMC3994071; doi:10.1371/journal.pone.0095586)
Supplement: Methods S1 — (DOCX) [file pone.0095586.s006.docx]

**Supplementary methods**

- **Immunocytochemistry**Subconfluent orbital control (C02-4) and GO (HO1-3) fibroblasts were trypsinised and fixed for 20 minutes with 3% formalin (Sigma), and then smeared on microscopy slides using cytospin. The slides were processed using Leica BOND-MAX™ automated IHC and ISH stainer and Bond Polymer Refine Detection kit (Leica) with addition of primary antibodies against either vimentin, factor VIII (both Dako) or pan cytokeratin (Leica), and the signal was developed using 3,3' Diaminobenzidine (DAB). Images were captured using light microscopy (Leica DMIL/Nikon DS-Fi1, 40X objective).

**CD45 FACS analysis**

Subconfluent orbital control (C02-4) and GO (HO1-3) fibroblasts were trypsinised, washed, and a minimum of 300,000 cells per condition were transferred into 15ml tubes. Cells were centrifuged and the pellet re-suspended in 100μl of either PBS or PBS with PE-conjugated anti-human CD45-RO antibody (Biolegend). Cells were kept on ice for 1 hour with occasional mixing of the tube, washed twice with PBS, re-suspended in 500μl of PBS, transferred to a FACS tube (BD Falcon polystyrene 352052) and analysed on a FACSCalibur (Becton Dickinson).

**Cell viability assay**

Control CO4 and GO HO1 fibroblasts were seeded in attached collagen gels as per the standard 3D adipogenesis protocol with 0 or 28 mmHg applied at day 5. The LIVE/DEAD cytotoxicity assay (Thermo Fischer Scientific/ Life Technologies) was used at day 7 according to manufacturer’s instructions, using 4μM ethidium homodimer-1 and 2μM calcein AM. Live cells (green) and dead cells (red) were then visualised in gels using confocal microscopy (Zeiss Axiovert S100/Biorad Radiance 2000, 10X objective). Images were acquired from 10 separate regions per gel, and the merged red/green images were used to count the percentage of Live and Dead cells.

**Western blotting**

HO2 GO cells were starved overnight in serum free medium. The PP2 SFK inhibitor (20 uM, Tocris Bioscience) was added to the medium and the cells were incubated for 30 min before stimulation with 15% serum for 5 or 30 min. The cells were then lysed with RIPA buffer supplemented with PhosSTOP Phosphatase Inhibitor Cocktail Tablets (Roche) and cOmplete, Mini Protease Inhibitor Tablets (Roche). The lysates were run onto 12% Precise ^TM^ Protein Gel (Thermo Scientific), and transferred onto PVDF membranes (Thermo Scientific). The membrane was blocked with 5% BSA (Sigma) in TBST, and the proteins were detected using Src[pY416] rabbit monoclonal antibody (Cell Signalling Technology) and GAPDH rabbit polyclonal antibody (Abcam), followed by HRP –conjugated goat anti-rabbit IgG (Abcam). The membranes were developed using Pierce ^TM^ ECL 2 Western Blotting Substrate (Thermo Scientific).

**Hyaluronic acid measurements**

HO1 cells were grown in 24 well plates until confluent. Confluent wells were serum starved overnight in medium with 1% serum, and treated with recombinant (rIGF-1; 10nM/L) with/without anti-IGF-1R antibody (1H7; 5ug/ml), as previously described [9]. After 48 hours the media were removed, and the cells were washed, and solubilised with NaOH by scraping the bottoms of the plates. The media and cell remnants were centrifuged and the supernatant transferred to an ELISA plate (Corgenix, Broomfield, CO) for hyaluronic acid (HA) quantification. The values were normalised to the cell numbers determined using an Alamar Blue Assay.
